# Tackling Immune Targets for Breast Cancer: Beyond PD-1/PD-L1 Axis

**DOI:** 10.3389/fonc.2021.628138

**Published:** 2021-03-05

**Authors:** Yasser Tabana, Isobel S. Okoye, Arno Siraki, Shokrollah Elahi, Khaled H. Barakat

**Affiliations:** ^1^Faculty of Pharmacy and Pharmaceutical Sciences, University of Alberta, Edmonton, AB, Canada; ^2^School of Dentistry, University of Alberta, Edmonton, AB, Canada; ^3^Department of Oncology, University of Alberta, Edmonton, AB, Canada; ^4^Department of Medical Microbiology and Immunology, University of Alberta, Edmonton, AB, Canada

**Keywords:** breast cancer, immunotherapy, therapeutic agents, immune targets, PD-1, PD-L1

## Abstract

The burden of breast cancer is imposing a huge global problem. Drug discovery research and novel approaches to treat breast cancer have been carried out extensively over the last decades. Although immune checkpoint inhibitors are showing promising preclinical and clinical results in treating breast cancer, they are facing multiple limitations. From an immunological perspective, a recent report highlighted breast cancer as an “inflamed tumor” with an immunosuppressive microenvironment. Consequently, researchers have been focusing on identifying novel immunological targets that can *tune up* the tumor immune microenvironment. In this context, several novel non-classical immune targets have been targeted to determine their ability to uncouple immunoregulatory pathways at play in the tumor microenvironment. This article will highlight strategies designed to increase the immunogenicity of the breast tumor microenvironment. It also addresses the latest studies on targets which can enhance immune responses to breast cancer and discusses examples of preclinical and clinical trial landscapes that utilize these targets.

## Introduction

The Global Cancer Statistics (GLOBOCAN 2018) report of 2018 flags breast cancer as the second most diagnosed cancer, with a prevalence of ~11.6% of all cancer cases (1). Breast cancer is the first diagnosed cancer and the leading cause of death among women, with over 450,000 mortalities annually (2). Based on the status of the tumor receptors, three types of breast cancers have been reported: estrogen/progesterone receptor-positive (ER+), human epidermal growth factor receptor 2-positive (HER2+), and triple-negative (TNBC) breast cancer (3). ER+ breast cancer is the most diagnosed breast cancer, with an incidence rate of ~80% (4, 5). Recently, the reactivation of the immune system has emerged as a strategy for cancer treatment other than traditional methods (6). Due to the immunological quiescent nature of breast tumors, immunotherapy has not been considered as a strategy for breast cancer treatment. However, this strategy has been reconsidered following the identification of tumor immune infiltrates. Since tumor-infiltrating lymphocytes (TILs: CD8+ cytotoxic T cells and helper CD4+ cells, regulatory T cells, B cells, NK cells), tumor-associated macrophages and myeloid-derived suppressor cells (MDSCs) are observed in some breast tumors (7, 8). Hence, the alteration and manipulation of the immune responses are now the focus of breast cancer therapeutic strategies (9). The discovery of inhibitory immune checkpoints has revolutionized cancer treatment (10). Understanding their role in promoting immunosuppression in the tumor microenvironment (TME) has resulted in the use of checkpoint inhibitors (generally monoclonal antibodies), which can reactivate immune cells (11, 12). Checkpoint inhibitors that target PD-1 or CTLA-4 have been used for treating metastatic breast cancer (13). However, the response rates were lower than other types of cancers; the overall response rate to anti-PD-1 (Pembrolizumab) was only 18.5% when used as monotherapy for patients with advanced triple-negative breast cancer (TNBC) (14). However, the KEYNOTE 355 study was initiated in 2016 to compare the effectiveness of using pembrolizumab in combination with chemotherapy with placebo plus chemotherapy for treating patients with unresectable locally advanced or metastatic PD-L1-positive TNBC (ClinicalTrials.gov Identifier: NCT02819518). Reports from this study indicated that pembrolizumab combined with several chemotherapy agents showed a statistically significant and clinically meaningful improvement in progression-free survival with 9.7 months vs. only 5.6 months with using chemotherapy alone in these patients. Pembrolizumab combined with chemotherapy showed adverse event rates 68% while 67% with chemotherapy. This combination was generally well-tolerated, with no safety concerns (15, 16). Based on the results of this trial, the FDA approved the use of pembrolizumab (anti-PD1) in combination with chemotherapy for the treatment of unresectable locally advanced or metastatic PD-L1-positive TNBC, in November 2020.

Nevertheless, identifying novel targets and developing new therapeutic agents are needed for breast cancer treatment. Other therapeutic targets that can modulate immune responses against breast tumors are currently under investigation. Co-stimulatory receptors are promising targets, which can improve anti-tumor immunity in breast cancer (13). Purinergic ectoenzymes attenuate the immune response by increasing the level of extracellular adenosine, which has immunosuppressive properties (17, 18). Inhibiting purinergic ectoenzymes will increase the anti-tumor immune responses (19). Similarly, targeting the immunosuppressive enzyme arginase 1 (ARG1), could also improve anti-tumor immune responses (20, 21). Studies have shown that various cytokines, chemokines, growth factors, and their receptors such as vascular endothelial growth factor (VEGF), VEGF Receptor (22), CXC receptor 1(CXCR1), CCL2 receptor (CCR2) (23), colony-stimulating factor-1 (CSF-1) (24) and toll-like receptors (TLRs) (25) are essential for breast tumor proliferation and metastasis. Furthermore, studies on targeting tryptophan catabolism enzymes, such as indoleamine-2,3-dioxygenase (IDO1/IDO2), and tryptophan-2,3-dioxygenase (TDO/TDO2), which are expressed by many immune cells and solid tumors, including breast cancer are underway (26). Moreover, the development of agents, which can modulate the COX2/PGE2 (27) and STING (28) signaling pathways, are ongoing.

**Figure 1:**
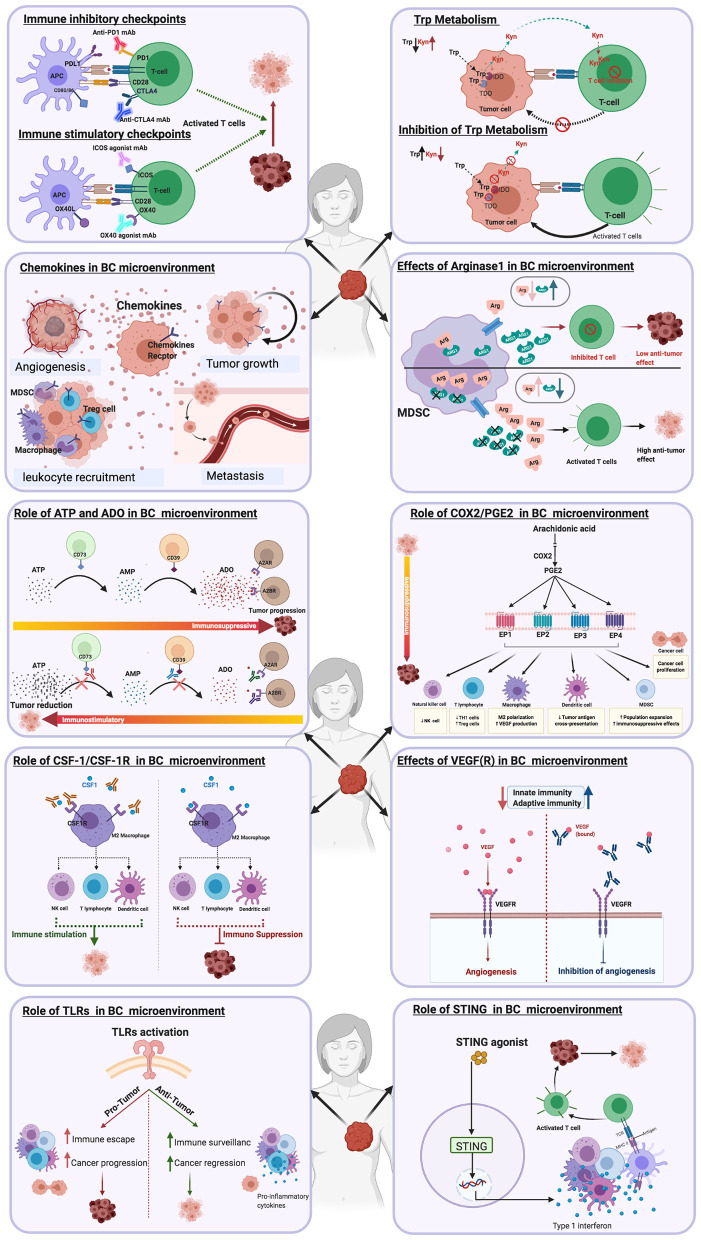
Immune targets in breast cancer immunotherapy.

The effects of blocking different immune checkpoints in breast cancer have been recently reviewed by Swoboda A, and Nanda R (29). Furthermore, the effectiveness of combining PD1/PD-L1 blockade with chemotherapy, targeted therapies and radiotherapy for the treatment of metastatic breast cancer has been reviewed by Page et al. (30). In this review, we will discuss the pathways that modulate immune responses to breast cancer (Figure 1). We will also discuss novel therapies and clinical trials designed to target these pathways (Table 1).

**Table 1:** Examples of clinical trials of Immune targets in breast cancer immunotherapy.

**Target**	**Drugs**	**Company**	**With combination**	**Phase**	**Clinicaltrials.gov identifier (selected trials)**
Anti-(PD-1)	Pembrolizumab	Merck	+Nab-paclitaxel/Paclitaxel/ Gemcitabine/Carboplatin	III	NCT02819518
Anti-(PD-L1)	Atezolizumab	Genentech/Roche	–	I	NCT01375842
			+ Nab-paclitaxel	III	NCT02425891
	Avelumab	Merck	–	III	NCT02926196
Anti-(CTLA-4)	tremelimumab	AstraZeneca	+Exemestane/ durvalumab	II	NCT02997995
			+ Durvalumab	I/II	NCT01975831 NCT02536794
	Ipilimumab	Bristol-Myers Squibb (BMS)	+ Nivolumab/ cobimetinib	I/ II	NCT01928394
			+ Enoblituzumab	I	NCT02381314
Anti-(LAG-3)	IMP321/Eftilagimod alpha	Immutep	+Paclitaxel	II	NCT02614833
Anti-(TIM-3)	MBG453	Novartis	+ Spartalizumab	I/II	NCT02608268
OX40 agonists					
	GSK3174998	GlaxoSmithKline	Alone/ with Pembrolizumab	I	NCT02528357
	MEDI-0562	MedImmune	–	I	NCT02318394
	MEDI-6383 (OX40L-Fc)	MedImmune	Alone/with MEDI-4736	I	NCT02221960
	PF-04518600	Pfizer	Alone/with PF-05082566	I	NCT02315066
	MEDI-6469	MedImmune	+Radiation	I	NCT01862900
	BMS-986178	Bristol-Myers Squibb	Alone/ with nivolumab ± ipilimumab	I/II	NCT02737475
	ABBV-368	Idera Pharmaceuticals	+ABBV-927 ± budigalimab	I	NCT03893955
GITR agonist					
	INCAGN01876	Incyte	Nivolumab and/ or ipilimumab	I/II	NCT03126110
	INCAGN01876	Incyte	Pembrolizumab and/ or epacadostat	I/II	NCT03277352
	TRX518	Leap Therapeutics	+ Cyclophosphamide +/or Avelumab	I/II	NCT03861403
4-1BB agonist	PF-05082566 (Utolimumab)	Pfizer	+ Trastuzumab – Emtansine	I	NCT03364348
	PRS-343	Pieris Pharmaceuticals, Inc. (PIRS)	+Atezolizumab	Ib	NCT03650348
CD40 agonist	CDX-1140	Celldex Therapeutics	Alone or with Pembrolizumab	I	NCT03329950
ICOS agonist	JTX-2011	Jounce Therapeutics	Nivolumab/Ipilimumab/ Pembrolizumab	I/II	NCT02904226
IDO1 inhibitor					
	Indoximod	NewLink Genetics	-	I	NCT00739609
			+Docetaxel/paclitaxel	II	NCT01792050
	Epacadostat	Incyte *Corporation*	+ INCMGA00012 and Epacadostat	I/II	NCT03328026
			+/or Itacitinib with INCB050465	I	NCT02559492
Targeting Arginase-1	Arginase-1 peptide vaccine	IO Biotech ApS.		I	NCT03689192
CXCR4 antagonist	balixafortide	Polyphor	+Eribulin	III	NCT03786094
CCR5 antagonist	Leronlimab	CytoDyn, Inc.	-	-	NCT04313075
CD73 antagonists	±Oleclumab (*MEDI9447*)	IMFINZI®	+ Carboplatin + Paclitaxel +Durvalumab	I/II	NCT03616886
	CPI-006	Corvus Pharmaceuticals	Alone/ with Ciforadenant +Pembrolizumab	I	NCT03454451
A2AR antagonist	CPI-444 Ciforadenant	Corvus Pharmaceuticals	Alone/ with Combination+ Atezolizumab	I	NCT02655822
PGEP4R blocker	AAT-007	Applied Therapeutics	-	II	NCT02538432
CSF1R blocker	LY3022855	Imclone Llc	Alone/ with Durvalumab or Tremelimumab	I	NCT02718911
	Pexidartinib (PLX-3397)	*Daiichi Sankyo*	+ Eribulin	I/II	NCT01596751
	Emactuzumab (RG7155)	Roche	+Atezolizumab		NCT02323191
			+RG7876	I	NCT02760797
VEGFR blocker	Ramucirumab	Eli Lilly and Company	+Docetaxel	III	NCT00703326
	Lucitanib	Clovis Oncology, Inc.	–	II	NCT02202746
TLR7 agonist	852A	Pfizer	–	II	NCT00319748
	Imiquimod	NYU Langone Health	–	II	NCT00899574
STING agonist	ADU-S100 (MIW815)	Novartis Pharmaceuticals	+Spartalizumab	I	NCT03172936
	E7766	Eisai Inc.	-	I	NCT04144140

## Stimulatory Checkpoints

**Figure 2:**
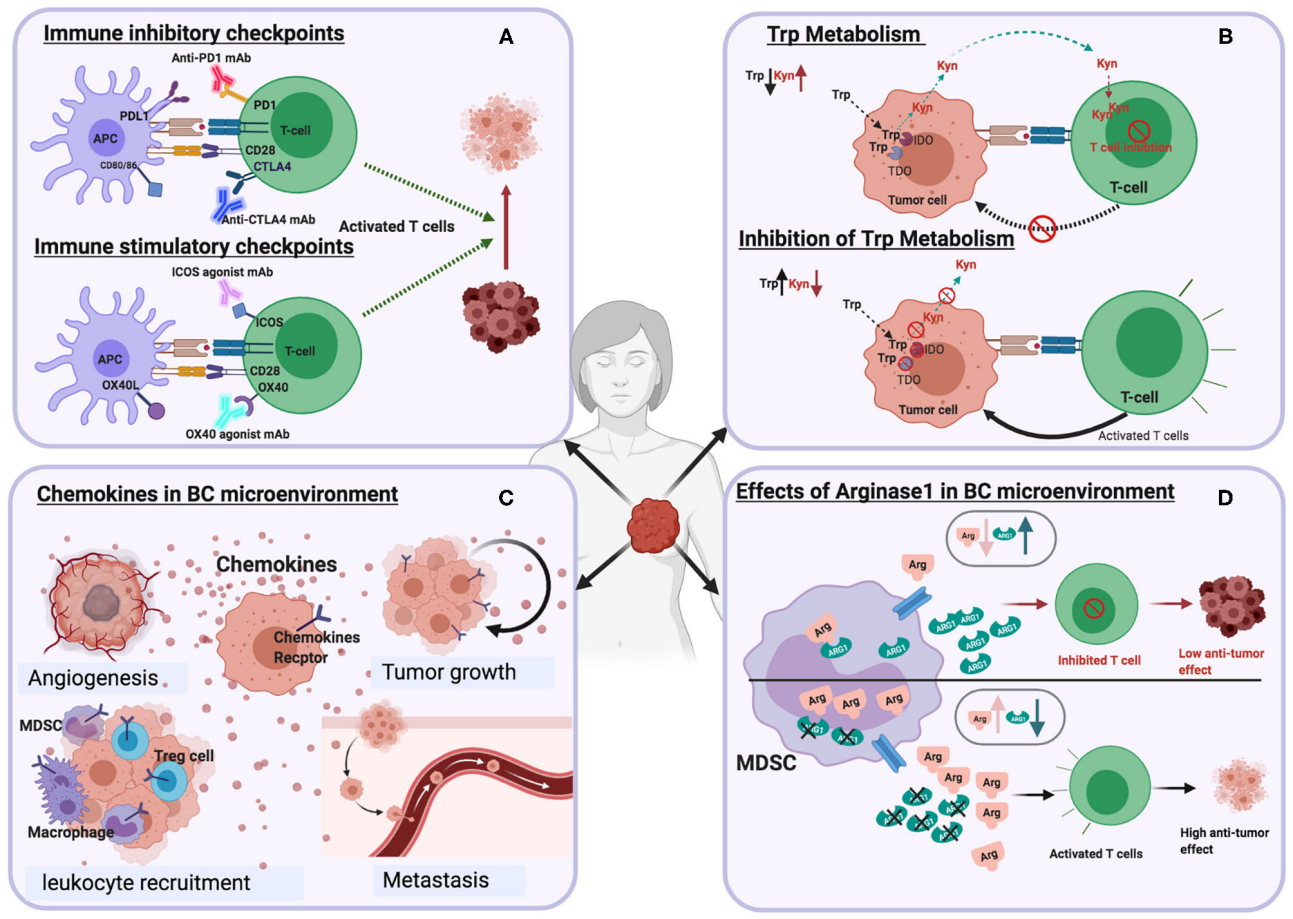
Schematic illustrations depicting the effects of different immune targets on breast cancer **(A)** Immune checkpoints **(B)** Tryptophan metabolism **(C)** Chemokines **(D)** Arginase enzyme.

A major characteristic of tumors is the paucity of, or ability to downregulate the expression of co-stimulatory molecules and upregulate co-inhibitory receptor expression (31, 32). The ligation of co-stimulatory molecules expressed by antigen-presenting cells (APCs) with their receptors on T cells provides the second signal necessary for T cell activation and differentiation. Hence, the use of co-stimulatory molecule agonist antibodies, is a strategy which may enhance T cell function in the TME (31, 32) (Figure 2A). Targeting co-stimulatory molecules that belong to the tumor necrosis factor receptor (TNFR) family such as OX40, ICOS, GITR, CD40L, and 4-1BB with agonist antibodies have been found to improve T cell function, with favorable outcomes in some cancer patients [reviewed in Moran et al. (33)].

OX40 (i.e., CD134) is expressed by TILs in various types of cancers, including breast cancer (34), while its receptor OX40L, is upregulated on monocytes, neutrophils, macrophages and dendritic cells. Studies have shown that OX40–OX40L signaling reduces immunosuppression mediated by regulatory T cells (Tregs) and enhances the expansion and proliferation of T cells (34). A study to assess the safety and tolerability of the OX40 agonist (PF-04518600) alone, or in combination with the 4-1BB agonist, PF-05082566, in patients with metastatic carcinoma, including TNBC was concluded in December 2020 (ClinicalTrials.gov Identifier: NCT02315066), (35). However, a clinical study that had planned to test the agonistic anti-OX40 antibody, MEDI6469, in combination with immune checkpoint inhibitors in patients diagnosed with advanced solid tumors, was terminated (32, 35, 36). Another phase I/II study, which investigated the use of MEDI6469 in combination with radiation for the treatment of metastatic breast cancer has been completed (ClinicalTrials.gov Identifier: NCT01862900). An additional phase I study has been initiated to investigate the effectiveness of using a CD40 agonist, ABBV-927 plus OX40 agonist ABBV-368 in combination or without the PD1 inhibitor, budigalimab in patients with advanced solid tumors, including TNBC (ClinicalTrials.gov Identifier: NCT03893955). Observations from a recent study indicated that OX40 agonists enhanced the production of IL-2 by conventional TILs, which increases the proliferation of both tumor-infiltrating Tregs and conventional T cells. Hence, in contrast to what has been postulated by previous studies, Tregs retain their immunosuppressive abilities in response to OX40 agonist treatment. However, results from this study also indicate that Tregs acquire a Th1 phenotype (IFN-g and granzyme B production) in response to OX40 agonist treatment (37). These observations imply that OX40 agonist treatment may be more suitable for combination therapies for cancer treatment. The importance of investigating the sequence of administering monoclonal antibodies in combination treatments that include anti-PD1 and OX40 agonists has been highlighted by Messenheimer et al. (38). They showed that using a preclinical model of oncogene-driven mammary cancer that concurrent administration of anti-PD1 antibody and an OX40 agonist compromised tumor regression. In contrast, sequential administration of the OX40 agonist and anti-PD1 facilitated tumor elimination, which was dependent on CD4+ and CD8+ T cell responses (38). These results indicate that sequential, rather than simultaneous administration of OX40 agonists and anti–PD-1 can revert PD-1 resistance and improve responses to combination therapy. Consequently, one of the approaches in a Bristol-Myers Squibb (BMS) clinical study (39) involves exploring the effectiveness of sequentially administering an OX40 agonist, BMS-986178, anti-PD1 (Nivolumab), an allogeneic autophagosome-enriched vaccine, DPV-001 and cyclophosphamide in TNBC patients (ClinicalTrials.gov Identifier: NCT02737475).

Another co-stimulatory molecule, the inducible co-stimulator (ICOS), is mainly expressed by activated CD4+ and CD8+ T cells and constitutively by Tregs. ICOS binds to its ligand, ICOS-L (B7RP1), expressed by APCs, epithelial cells, endothelial cells and tumor cells (40). ICOS-mediated co-stimulation does not induce IL-2 production, hence it is regarded as less potent relative to co-stimulation elicited by CD28 (41, 42). However, various clinical studies have shown that high expression of ICOS by T cells in patients treated with PD-1 and CTLA-4 checkpoint inhibitors correlates with positive treatment responses (43, 44). Hence, current immunotherapy strategies include the administration of ICOS or ICOS-L agonists with CTLA-4 checkpoint inhibitors (43, 45). A Phase 1/2 first in-human clinical trial has been set up to evaluate JTX-2011, an agonist monoclonal antibody that binds to ICOS, alone or in combination with checkpoint inhibitors for the treatment of advanced solid tumors, including TNBC breast cancer (46). A recently completed phase 1 clinical trial, which involved the use of another ICOS agonist, GSK3359609, in combination with anti-PD-1 shows promising anti-tumor activity in anti-PD-1/L1 naive patients with head and neck squamous cell carcinoma (HNSCC) (32, 36). Furthermore, the findings from this study indicate that GSK3359609 is also suitable for monotherapy of HNSCC in patients with anti-PD-1/L1 -experienced HNSCC (GSK Press Release September 28, 2019).

The glucocorticoid-induced TNFR related protein (GITR) is preferentially expressed on NK cells and T cells, particularly Tregs. GITR interaction with its ligand, GITRL, on dendritic cells, boosts effector T cell differentiation and IL-2 production (11, 13). Importantly, GITR has been detected on lymphocytes and carcinoma cells from a subset of breast cancer tumor specimens (47). Furthermore, observations from a study by Krausz et al. indicated that Tregs from tumor-positive lymph nodes from advanced breast cancer patients express increased levels of GITR, compared to tumor-negative lymph nodes (48). The potential for GITR-mediated co-stimulation to promote high effector CD8+ T cell to Treg ratios, is now harnessed as an immunotherapy strategy (49, 50). In fact, the first in-human phase 1 trial of GITR agonism with the anti-GITR antibody TRX518, has been initiated and a report indicates reduction in circulating and intratumoral Tregs at similar levels (51). However, a combination of GITR agonism with PD-1 blockade has been postponed due to sub-optimal clinical responses induced by TRX518 (51, 52). A clinical trial using another anti-GITR agonistic mAb, INCAGN01876, in combination with pembrolizumab and epacadostat for the treatment of advanced or metastatic malignancies is underway (ClinicalTrials.gov Identifier: NCT03277352).

CD40 is upregulated on the surface of activated APCs and its interaction with its ligand (CD40-L), expressed on activated B cells and T cells, leads to the initiation and progression of cellular and humoral adaptive immunity (53, 54). CD40 is also expressed in breast and lung carcinomas and carcinomas of the urinary bladder, nasopharynx, and colon, in contrast to normal non-proliferating tissues, which are CD40-negative (55, 56). Observations from a study approximately two decades ago by Tong et al., indicated that the interaction of soluble recombinant CD40L with CD40+ human breast cancer cell lines directly inhibits breast cancer cell growth. By examining primary tumor biopsies, they also found that infiltrating ductal, lobular carcinomas and carcinomas expressed CD40 while benign epithelial tissues of these biopsies exhibited weaker expression of CD40 (57). Interestingly, tumor infiltrating lymphocytes from most of the breast cancers examined expressed very low levels of CD40L (57). Other studies have suggested that CD40 may induce apoptosis in breast carcinoma cells by upregulating Fas expression induced by CD40 ligation (58).

A clinical study of CDX-1140, a CD40 agonist, for use as a monotherapy or in combination with the anti-PD-1 mAB, pembrolizumab, has been initiated in patients with advanced malignancies, including breast cancer (ClinicalTrials.gov Identifier: NCT03329950). Furthermore, results from a recent orthotopic breast cancer study suggest that combination treatment using anti-PD-1 and a CD40 agonist promote tumor immunogenicity (59).

4-1BB (CD137) is another member of the TNFR family of co-stimulatory molecules. It is expressed on many hematopoietic cells, including T cells and NK cells. Its ligand, 4-1BBL (CD137L), is predominantly expressed on APCs. 4-1BB:4-1BBL ligation potentiates CTL responses, induces antibody-dependent cell-mediated cytotoxicity in NK cells and modulates the activity of CD4+ T cells, B cells, DCs, monocytes and macrophages (60). For instance, CD8+ TILs from TNBC tumors were successfully propagated with a 4-1BB agonistic antibody (urelumab) (61). Based on these properties, harnessing the 4-1BB signaling pathway through the use of agonistic monoclonal antibodies can serve as a cancer immunotherapy strategy.

Significant breast tumor reduction in xenograft models has been achieved by targeting 4-1BB, combined with trastuzumab (anti-HER2) and rituximab (anti-CD20) treatment (32, 62, 63). In 2017, a clinical trial to investigate the optimal dosage and side effects of the 4-1BB agonist, utomilumab with trastuzumab emtansine or trastuzumab in patients with metastatic HER2-positive breast cancer was initiated (ClinicalTrials.gov Identifier: NCT03364348). However, a dependency of 4-1BB agonists on the Fcγ receptor–mediated hyperclustering and liver toxicity in patients, have been reported (64). Consequently, strategies that will restrict 4-1BB agonism to the TME, thereby minimizing off-target toxicities, have been proposed. A recent study has adopted a protein engineering approach to develop proteins that simultaneously target 4-1BB and tumor stroma or tumor antigens (65).

## Amino Acid Catabolism

Amino acid metabolism is an immune regulatory mechanism (52). The breakdown of amino acids, particularly tryptophan and arginine by immunoregulatory myeloid cells, is one mechanism whereby T cell proliferation and activation are suppressed (29). Furthermore, these catabolic pathways are harnessed by solid tumors to induce the development of immunosuppressive tumor microenvironments and poor anti-tumor T cell responses. Hence, the use of inhibitors of arginase-1 and indolamine- 2, 3- dioxygenase-1 enzymes, which catabolise L-arginine and tryptophan, respectively, are now exploited as new cancer immunotherapy strategies.

### Catabolism of Tryptophan

Tryptophan is the rarest essential amino acid found in food. It is a precursor to the synthesis of niacin (vitamin B3), neurotransmitter serotonin, and the hormone melatonin. Tryptophan metabolism is associated with immune regulation and tumor progression (66). Tryptophan catabolism occurs through the kynurenine pathway with the aid of two enzymes, indoleamine-2,3-dioxygenase (IDO1) and tryptophan-2,3-dioxygenase (TDO), which catalyze the first rate-limiting step by facilitating the oxidative breakdown of the tryptophan indole group. The generation of kynurenine (Kyn) and the concomitant release of kynurenine metabolites by myeloid cells, suppresses T cell and NK cell activity. The activities of IDO and TDO have been investigated due to their link with various diseases, including diabetes, mental disorders, inflammatory, and cancer (67, 68) (Figure 2B).

#### Indoleamine-Pyrrole 2,3-Dioxygenase (IDO1)

The upregulation and sustained expression of IDO by tumor cells is a well-characterized immunosuppressive strategy, orchestrated in conjunction with MDSCs and Tregs (69). IDO1 and TDO, through their catalytic activity, function as tryptophan *sinks*, leading to the suppression of T cell proliferation, apoptosis and Tregs differentiation. Indeed, T cell activation and function are highly dependent on the levels of tryptophan in their microenvironment, as the zeta chain of TCR complex is downregulated upon tryptophan withdrawal. IDO1 also suppresses anti-tumor responses through the generation of L-kynurenine, an endogenous agonist of the arylhydrocarbon receptor (AhR). AhR activation promotes the differentiation of Tregs and the concomitant upregulation of IDO1 by DCs (70). Furthermore, long-term expression of IDO1 by DCs is facilitated when IDO functions as a signal-transducing molecule (70).

The expression of IDO has been observed in breast carcinomas, particularly among triple negative (TNBC) basal-like breast cancers (71, 72). In a study by Dill et al., the authors assessed 281 primary and metastatic breast cancers and identified a correlation between IDO1 and PD-L1 expression, particularly in high-grade TNBC (73). Their observations imply that IDO1 expression contributes to the resistance of breast cancer to anti-PD-1/PD-L1 treatment.

A positive correlation between the high expression of PD-1 by T cells and high levels of kynurenine in the plasma and the TME of breast cancer patients has also been reported (74). IFN-γ produced by CD8+ T cells induces the production of IDO and kynurenine by CD45 negative tumor cells. Kynurenine promotes the translocation of AhR from cytosol to the nucleus of *in vitro-*treated and tumor-infiltrating CD8+ T cells and subsequently upregulates PD-1 (60).

IDO1 also induces cancer progression in a non-immune manner by regulating angiogenesis (59). The expression of IDO and levels of CD105+ micro vessel density by breast cancer specimens were found to be associated with metastasis and poor prognosis (75). Furthermore, MCF-7 cells which produce high levels of IDO significantly induced the proliferation of human umbilical vein endothelial (HUVEC) cells (75). Thus, the pharmacological modulation of IDO1 and other enzymes that target amino acids have been included in cancer therapy strategies (20). Preclinical and clinical studies to test the efficacy of IDO inhibitors for cancer treatment are discussed extensively in a recent review (76).

A number of studies in which IDO1 is targeted alone or in combination with immune checkpoint inhibitors have been proposed. In 2017, a phase II clinical study investigated the effect of the combined use of chemotherapy and the IDO1 inhibitor, 1-Methyl-D-tryptophan (Indoximod) in metastatic breast cancer patients (ClinicalTrials.gov Identifier: NCT01792050). Results from the phase I study indicated no drug-drug interactions and partial responses in breast cancer and patients with other metastatic tumors (77). Four of the breast cancer patients achieved a reduction in tumor burden; a patient that had hitherto only received only adjuvant endocrine therapy achieved the best response (77). Results from another phase I study on the use of a small molecule inhibitor of IDO1 (Navoximod) alone, or in combination with a PD-L1 inhibitor (Atezolizumab) to treat TNBC and other solid tumors indicated tolerability, partial responses and complete responses in some patients (78). However, there were no clear benefits associated with the use of atezolizumab with navoximod (78). Results from another phase I/II study of another IDO inhibitor, Epacadostat, used in combination with anti-PD-1 (pembrolizumab) for the treatment of TNBC and ovarian cancer indicated tolerability, safety and anti-tumor activity (79). However, in another study, there was no difference in progression-free or overall survival in patients with unresectable stage III or IV melanoma administered with Epacadostat in combination with anti-PD1 (pembrolizumab), compared to placebo plus pembrolizumab (80). Hence, the usefulness of IDO1 inhibition as a strategy to enhance anti-PD-1 therapy activity in cancer yet to be clarified.

Other approaches which utilized nanodelivery systems designed to use Indoximod in conjunction with a-PD-L1 or the induction of immunogenic cell death using doxorubicin for breast cancer treatment, have also been investigated (81). Taken together, the outcomes of these studies suggest that IDO1 inhibitors can be used as standard-of-care treatment for breast cancer and other solid tumors, alone or in combination with other cancer therapeutic strategies.

#### Tryptophan-2,3-Dioxygenase (TDO)

Unlike IDO1, which is induced in immune cells such as DCs, TDO is constitutively expressed in the liver, where it regulates tryptophan homoeostasis in the blood (82–84). Similar to IDO1, TDO suppresses T cell activation by tryptophan depletion and is also overexpressed in the microenvironment of various tumors, including breast cancer (26). Preclinical studies have demonstrated that TDO expression by breast cancer cells is associated with increased cancer cell migration and invasion (66, 85). In a study by Greene et al., the authors demonstrated that triple-negative breast cancer (TNBC) cells use TDO to suppress CD8+ T-cell viability (86). Furthermore, in an earlier preclinical study, D'Amato et al., showed that NF-kB-dependent upregulation of TDO and AhR is linked to anchorage-independent cell survival and anoikis resistance of TNBC cells (85). These observations imply that the overexpression of TDO by tumors such as TNBC is associated with disease metastatic.

Results from preclinical studies investigating the impact of TDO inhibition using knockout mice or compounds have shown that deletion of the TDO gene (*TDO2*) in mice results in tryptophan accumulation in the blood and neurologic changes, which may be associated with serotonin production (84) Consequently, the utilization of TDO inhibitors may have safety implications with respect to liver and CNS complications. Dose-dependent reduction of the 4T1 breast or CT26 colon tumor growth was achieved by dual inhibition of IDO and TDO using a lead compound, CB548, in a mouse preclinical model (87). Also, the administration of CMG017, another dual inhibitor of IDO and TDO, to tumor-bearing mice resulted in reduced kynurenine concentration, differential expression of immune-related genes and the infiltration of effector CD8+ T cells in the TME (87). Furthermore, co-administration of CMG017 with checkpoint inhibitors (a-PDL1 and a-CTLA-4) to tumor-challenged mice resulted in tumor regression and the establishment of memory CD8+ T cell responses (87).

In 2017, a phase I study was initiated to investigate the safety, pharmacokinetics, pharmacodynamics and efficacy of HTI-1090, a small molecule dual inhibitor of IDO1 and TDO, in patients with advanced solid tumors (ClinicalTrials.gov Identifier: NCT03208959). Although this study was completed in 2019, the outcomes are yet to be disclosed. The utilization of other TDO and IDO1 inhibitors such as 680C9, LM101 are still under preclinical investigation.

### Catabolism of Arginine

#### Arginase

L-arginine is a non-essential amino acid that plays a vital role in cellular activity such as metabolic programming and maintenance of T cell fitness (88, 89). The administration of L-arginine to breast tumor-bearing BALB/c mice suppressed tumor growth significantly and prolonged the survival time of treated mice. L-arginine supplementation also enhanced the levels of IL-10, TNF-α, IFN-γ; macrophage and T cell numbers and suppressed the activity of MDSCs. The activity of arginase enzymes (ARG1 and ARG2), which catalyze L-arginine into ornithine and urea, is increased in the TME of multiple cancers including breast cancer. Arginase enzymes facilitate localized immune suppression mediated by cancer-associated fibroblasts (ARG2), MDSCs, DCs, tumor-associated macrophages (TAMs) and tumor-infiltrating macrophages (ARG1) (90, 91). These cells in turn, produce ARG1 in response to a milieu of tumor cues, such as HIF-1α, M-CSF, GM-CSF, IL-4, IL-13 and IL-6 (89). Another key enzyme associated with L-arginine metabolism, nitric oxide synthase (NOS), produces nitric oxide (NO) from L-arginine and oxygen. In low L-arginine conditions, characteristic of tumor sites, NOS can induce the production of superoxide anion, which can combine with NO to generate various reactive nitrogen species that can also hamper T cell activity at tumor sites (89).

The reduction of extracellular arginine by ARG1 leads to suppression of T cell function (Figure 2D) by the activation of GCN2 kinase, which blocks the expression of several cell cycle genes such as cdk4, cyclin D3, and CD3 (21). High levels of ARG1 have been identified in the serum of preoperative breast cancer patients compared to healthy controls (92). In addition, elevated ARG1 is expressed by MDSCs from patients diagnosed with early-stage breast cancer, which is reduced upon surgical tumor resection (2).

A number of preclinical strategies that target ARG1 have been implemented with promising results. The cell viability and arginase activity of a TNBC cell line with high levels of arginase (MDA-MB-468), were decreased in response to L-lysine induced arginase inhibition, in comparison to a cell line with less arginase levels (MDA-MB-231) (93). The treatment of tumor bearing mice (CT26, 4T1, B16, and LLC) with CB-1158, a small molecule inhibitor of ARG1, elicited increased cytotoxic T cell infiltration and decreased myeloid cell numbers (71). This correlated with increased activation markers, cytokine production and expression of interferon genes. Furthermore, CB-1158 efficacy was enhanced when combined with checkpoint blockade, chemotherapy and adoptive cell therapy (94).

Treatment with the arginase inhibitor (INCB001158) alone inhibited plasma arginase activity with concomitant increase in the plasma arginine in a colorectal carcinoma patient cohort. INCB001158 used in combination with a-PD-1 (pembrolizumab) for the treatment of advanced/metastatic solid tumors. INCB001158-pembrolizumab combination treatment elicited increased frequencies of intratumoural CD8+ T cells and a 7% partial response (ClinicalTrials.gov Identifier: NCT02903914). A clinical study has been initiated to evaluate the safety, toxicity and immune correlates of administering an Arginase-1 peptide vaccine (ARG1-18,19,20) to patients with breast cancer and other solid tumors (ClinicalTrials.gov Identifier: NCT03689192).

## Chemokines and Chemokine Receptors

Chemokines and their receptors play a pivotal role in various biological and pathological processes, including chronic inflammation, tissue development, hematopoiesis, and immune modulation (95). Many studies revealed chemokines' role as essential mediators of immunity, angiogenesis (96), metastasis (97), drug resistance (98), breast cancer occurrence and progression (Figure 2C) (23, 99, 100). Chemokines have been classified into four main groups, CXC, CC, XC, and CX_3_C. The CXC family consists of 17 subfamily members (CXCL1-CXCL16), while CC family is the largest subgroup (CCL1-CCL28). The XC family has two subgroups (XCL1 and XCL2), while there is only one CX_3_C chemokine (CX_3_CL1) (95, 101).

Tumor cell migration and the ensuing invasion into specific organs occur in response to receptor-ligand interactions, the rearrangement of the actin cytoskeleton and multiple environmental cues which favor trafficking. Mueller et al., in investigating the role of chemokine receptors in promoting breast cancer metastasis almost two decades ago, found that breast cancer cells express CXCR4 and CCR7 (90). Consequently, targeting chemokines and their receptors has been evaluated in preclinical and clinical cancer immunotherapy studies. The detailed roles of chemokines in cancer biology have been reviewed elsewhere (23, 95, 102). We will highlight a few examples of the roles of chemokine–chemokine receptor interactions in the breast cancer microenvironment.

### CXCR Family

CXCL8 (IL-8) is a chemokine whose physiological effects are mediated by two receptors, namely CXCR1 and CXCR2 (103). CXCR2 (IL-8 receptor) is expressed on MDSCs, neutrophils, lymphocytes, and breast cancer cells. CXCR2 and CXCL8 regulate breast cancer progression in the TME by modulating several related pathological processes, including promoting breast cancer growth, angiogenesis, invasion, metastasis, and reducing cancer cell sensitivity to chemotherapy (99, 104, 105). CXCR2 modulates the trafficking of neutrophils from the bone marrow to breast cancer sites, leading to increased tumor growth (106). CXCR2 also induces the migration of MDSCs, thus, promoting local immunosuppression (107). Studies show that cancer patients with high levels of CXCR2 have low overall survival and poor prognosis (108). The CXCL8-CXCR2 axis can also stimulate the transcription of VEGF and activate its receptor,VEGFR2, in endothelial cells by the NF-κB pathway (109). Like CXCR2, CXCR1 is expressed significantly in breast cancer stem cells, which increases the growth of breast cancer when stimulated by inflammation or tissue damage (110). Consequently, targeting the CXCL8-CXCR1/CXCR2 axis has been adopted as a breast cancer therapy strategy (111). The utilization of reparixin, a small molecular weight antagonist of CXCR1/2 as a breast cancer therapeutic agent has been investigated in preclinical and clinical studies (99, 112). Results from a phase Ib trial on the co-administration of reparixin and paclitaxel to patients with HER-2- negative metastatic breast cancer yielded a 30% response rate (88). In another study on the treatment of women with HER-2- negative operative breast cancer with reparixin only, the frequency of cancer stem cells, indicated by aldehyde dehydrogenase, CD44+/CD24- expression, was reduced (113).

Several studies have assessed the impact of CXCR4 in breast cancer cell survival, proliferation, angiogenesis, migration, and metastasis (114, 115). CXCR4 induces breast cancer metastases by binding to its ligand stromal cell-derived factor-1α (SDF-1), which is overexpressed in the bone marrow, liver, lung, and breast tumors sites (100, 116). CXCR4 promotes cancer cell proliferation by activating several signaling pathways, including Src/ERK1-2, PI3K/AKT, STAT3, and NF-κB. The cross-link between CXCR4 and other pathways such as Notch, Wnt, and SHH is also associated with increased breast cancer growth (117). Injecting immunocompromised mice subcutaneously with a CXCR4-low-expressing breast cancer cell line (MCF-7), resulted in reduced tumor growth compared to mice inoculated with the MDA-MB-231 cell line, which expresses high levels of CXCR4 (118). Also, results from a human study in which surgically resected ductal carcinomas were evaluated, indicate that high CXCR4 expression correlates with extensive nodal metastasis (119). Preclinical studies of CXCR4 inhibitors have demonstrated its ability to attenuate the proliferation and metastasis of breast tumors; AMD3100 is a CXCR4 antagonist that decreases lung metastases in breast cancer (120). However, Lefort et al., have shown that AMD3100 and TN14003, another CXCR4 inhibitor, impair only the growth and metastasis of HER2 breast cancers, but not TNBC (121).

In contrast to the preclinical outcomes, the efficacy of CXCR4 blockade in clinical trials has not shown clear success with respect to dosage and the manifestation of undesirable side effects. In a clinical study by Pernas et al., the safety, tolerability, pharmacokinetics, and preliminary phase 1 dose-escalation activity of the CXCR4 antagonist, balixafortide, in combination with eribulin (antineoplastic) chemotherapy, was assessed in patients with relapsed metastatic breast cancer who had hitherto received chemotherapy (96). Partial responses were observed and serious side effects occurred in 30 and 38% of the study patients, respectively. Furthermore, two patients died from septic shock and pneumonia, respectively (96). Based on the observations of the Phase 1 trial, a phase 3 study has been set up to investigate the safety, efficacy and tolerability of intravenous balixafortide administered with eribulin compared to eribulin monotherapy for the treatment of HER2 negative, locally recurrent or metastatic breast cancer patients (ClinicalTrials.gov Identifier: NCT03786094).

### The CCR Family

CCL2 is overexpressed in tumor cells, including breast, ovarian, and lung cancer. CCL2 stimulates the migration of macrophages that express the chemokine CCL2 receptor (CCR2), into the TME. It also induces cancer proliferation and invasion (122). CCL2 can induce the migration of various breast cancer cell lines, including T47D, MCF-7, and ZR-75-1 (123). Studies using breast tumor xenografts show that blocking CCL2-CCR2 axis suppresses the recruitment process of inflammatory monocytes, increases tumor growth, and promotes metastasis and invasion (124). These studies suggest that CCL2-CCR2 signaling promotes breast cancer progression, and targeting this pathway might be adopted as a breast cancer therapy strategy.

CCL5/CCR5 pathway also plays a critical role in promoting breast cancer progression. CCL5 ligand is overexpressed in breast cancer cells, mesenchymal stem cells (MSCs), and infiltrating leukocytes. Results from a clinical study indicate that levels of CCL5 in breast cancer patients are higher than that of healthy controls (125). CCL5 can maintain the immunosuppressive activity of human MDSCs (126). The CCL5 receptor (CCR5) is also upregulated on breast cancer cells (127). A study conducted on breast cancer patients showed that 50% of breast tumors express CCR5, with >95% TNBC tumors being CCR5+ (128). The blockade of CCR5 suppresses breast cancer proliferation, migration, colony formation, and metastasis (129). Therefore, targeting CCR5 could be promising strategy for metastatic breast cancer. Met-CCL5, a competitive CCR5 inhibitor, reduces breast cancer proliferation and infiltrating macrophages in animal preclinical models (130). Treatment with maraviroc, CCR5 antagonist, significantly suppresses bone metastasis in a xenograft rat model implanted with breast cancer cells (MDA-MB-231) (131). Leronlimab (PRO 140) is another CCR5 antagonist under investigation in breast cancer clinical trials (129, 132, 133).

## Purinergic Signaling

**Figure 3:**
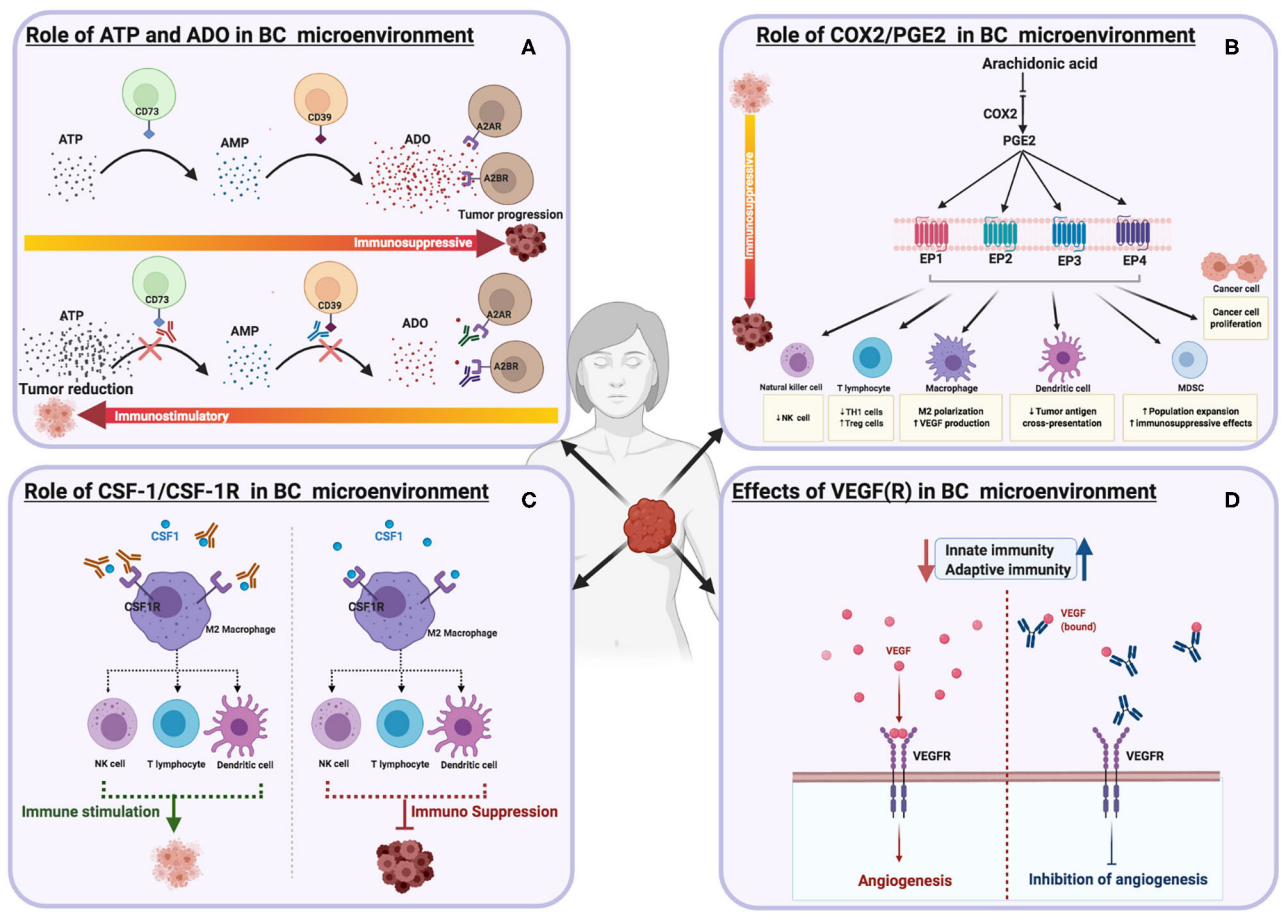
Schematic illustrations depicting the effects of different immune targets on breast cancer **(A)** ATP and Adenosine signaling **(B)** COX2/PGE2 pathways **(C)** CSF-1/CSF-1R **(D)** VEGF(R).

Purinergic signaling plays a prominent role in inflammation and cancer. It modulates cell growth, migration, and cell death (134). In this pathway, two potent molecules (ATP and Adenosine) involved in the immune response are released into the TME (Figure 3A) (135). Intracellular ATP levels are sustained at millimolar concentrations under physiological conditions, while extracellular levels are regulated in nanomolar concentrations. However, in the TME, ATP concentrations arise due to release from necrotic or apoptotic cells (136). Adenosine concentrations in solid tumors are also higher than that of healthy tissues (137, 138). It is well-reported that ATP and Adenosine have opposite effects. ATP is immunostimulatory as it enhances the activation of dendritic cells (DC), macrophages, IL-1β secretion, and cytotoxicity of CD8+ T cells. Hence, ATP activity can mediate the suppression of proliferating cancer cells. Adenosine, on the other hand, has immunosuppressive properties. It inhibits immune effector cells, DC maturation, cytokine production and stabilizes immunosuppressive Tregs (139). Purinergic cell surfaces ectoenzymes (P2Xs, P2Ys, CD73, CD39, and CD38), mediate the biological activities of ATP and Adenosine, and adenosine receptors (A1R, A2AR, A2BR, A3R), are overexpressed by breast cancer cells and tumor-infiltrating immune cells (19). Several therapeutic agents are developed to target these receptors to enhance anti-tumor immune responses against breast cancer.

### The P2 Family

The pyrogenic receptors P2Xs (ion channel receptors) and P2Ys (G protein-coupled receptors) are overexpressed on several immune cells within the TME (140). Among the pyrogenic receptors, P2X7 receptor (P2X7R) has been studied extensively due to its contrasting effects (134). In some studies the role of P2X7 in inducing antitumor immune responses by activating NK cells, CD4+, and CD8+ effector T cells, and promoting Treg apoptosis, has been shown (141, 142). Two P2X7 receptor agonists ATPγS and BzATP, reduce tumor growth and metastasis (143, 144). Other pieces of evidence propose the P2X7 receptor as promoters of tumor progression, mediated by inducing tumor growth, metastasis, and survival (145). P2X7R is upregulated in various tumors, including malignant breast cancers, and its expression is higher in tumors compared to the healthy tissue. This indicates that P2X7 can be used as an effective early cancer biomarker (40, 146). Many inhibitors that target P2X7R have been developed, such as Anthraquinone Emodin, which can potently suppress invasive breast cancer cells *in vitro* (147). AZ10606120 is another P2X7R antagonist reported to be a potent inhibitor of tumor growth (91).

### CD39 and CD73

ATP and ADP are converted into AMP by the catalytic activity of CD39, while AMP is irreversibly converted to adenosine by CD37 (148). CD39 and CD73 are expressed significantly by breast cancer and various immune cells, including T cells, NK cells, B cells, MDSC, macrophages, and neutrophils (17). The high expression of CD39 and CD73 results in increasing adenosine levels in the TME, which in turn stimulates the adenosine A2A and A2B receptors. The adenosine A2A and A2B receptors promote tumor progression by triggering angiogenesis, tumor cell survival, and metastasis (149–151). They also increase the immunosuppressive efficacy of Tregs, macrophages, MDSCs and development of effector T cells. Breast cancer patients with positive clinical outcomes exhibited low expression of CD39 and CD73 compared to patients with poorer clinical outcomes, which indicates that CD39 and CD73 can serve as biomarkers of patients' progress (152–154). Blocking CD73 and CD39 promoted anti-tumor responses; anti-CD73 mAbs, enhances the cytotoxicity of CD8+ T cells and inhibits the activity of Tregs and MDSCs (155). Small molecules against CD73 such as LaSOM 63 and APCP, inhibit tumor progression and increase the efficacy of effector T cells (150, 156). Preclinical studies indicate that anti-CD73 mAbs can hinder metastasis in human breast cancer (157). Three CD73 antagonists (MEDI9447, BMS-986179, CPI-006), which target TNBC are currently under clinical investigation (158). Similarly, preclinical studies of anti-CD39 monoclonal antibodies, BY40 and BA54G, have demonstrated anti-tumor efficacy (159). Therapeutic agents that target CD39 are still in the developmental stage (160).

### Adenosine A2A Receptor (A2AR) and Adenosine A2B Receptor (A2BR)

Extracellular adenosine stimulates the immunosuppressive pathway through engagement with specific G-protein-coupled adenosine receptors such as (A2a and A2b) (160). A2aR (high affinity receptor) is upregulated on a variety of immune cell subsets, including monocytes, macrophages, DCs, T cells, and natural killer T (NKT) cells. Adenosine signaling pathway through the A2aR suppresses T cell proliferation by increasing the expression of anti-inflammatory cytokines (IL-10, TGF-β) and reducing the expression of pro-inflammatory cytokines (IFN-γ, IL-2) (161). It also triggers increased expression of immune checkpoints such as LAG-3, PD-1, and CTLA-4 (162, 163). A2aR is overexpressed in many cancer cells, including breast cancer cells. Activation of A2aR leads to an increase in the proliferation of MCF-7 breast cancer cells (164). A2aR promotes proliferation and metastasis by stimulating various signaling pathways, including PLC/PKC, ERK-MAPK, PI3K/AKT/mTOR (165). CPI-444, an A2AR antagonist, is used as monotherapy or combined with anti-PD-L1 (Atezolizumab) to treat TNBC (166). A2bR, on the other hand, is a low-affinity receptor which needs more Adenosine to be activated. A2bR is overexpressed by macrophages, DCs, and multiple tumors such as breast tumors (167, 168). Its upregulation is associated with poor survival and worse prognosis in human TNBC (169). *In vitro* activation of A2bR, increases the growth and migration of breast cancer (MDA-MB-231) cells (170). Results from an *in vivo* study indicate that blocking A2bR reduces the metastasis of TNBC and enhances the activities of chemotherapy and immune checkpoint inhibitors (169). Several studies indicate that stimulating A2bR promotes tumor growth and metastasis through the activation of the ERK1/2 and angiogenesis pathways; blocking this receptor reverses these effects (19, 171, 172). A selective A2bR blocker (ATL801) promotes the inhibition of bladder and breast cancer growth when injected intratumorally (173).

## Targeting the COX2/PGE2 Pathways

Increased levels of COX2 enzymes have been reported in nearly half of breast cancer patients (174), with other studies reporting a range of 17 to 84% (175, 176). The silencing of COX-2 expressed by the human breast cancer cell line, MDA-MB-231, inhibits cell migration *in vitro* and metastasis *in vivo* (177). PGE2, an enzymatic product of COX2, functions by signaling through one of the four G-protein coupled receptors (EP1, EP2, EP3, and EP4) (Figure 3B). The COX2/PGE2 axis promotes breast cancer progression by increasing cancer migration, metastasis, and angiogenesis (178–180). In addition, PGE2 regulates different immune cells- it suppresses the proliferation of CD4+ T cells by reducing intracellular calcium release and suppressing the activity of the p59 protein tyrosine kinase (181, 182). PGE2 decreases the production of effector cytokines, such as IL-2 and IFN-γ, and it can also inhibit NK cell function and B cell proliferation (183–185). PGE2 elevates cAMP by the stimulation of its receptors, EP2 and EP4 (186). COX2/PGE2 and its receptors are potential target(s) for breast cancer therapy. Preclinical studies indicate that celecoxib, a selective COX-2 inhibitor, reduces breast cancer metastasis (176, 187). The daily intake of COX-2 inhibitors such as non-steroidal anti-inflammatory drugs (NSAIDs) reduce the risk of breast cancer occurrence significantly (188). The PGEP4 receptor blocker (AAT-007) is currently in phase 2 for the treatment of patients with solid tumors, including breast cancer (179). A newer version of the PGEP4 receptor antagonist called (AAT-008) has shown significant bioavailability and pharmacological profiles in preclinical investigations (189). The PGE2 EP1 antagonist (ONO-8711) suppresses breast cancer progression in rats (190). Using different breast cancer cell lines *in vitro*, the PGEP3 receptor antagonist (L798,106), demonstrated potency in reducing breast cancer proliferation and migrations (191).

## CSF-1/CSF-1R

Activated macrophages are divided, for simplicity, into anti-tumor (M1) macrophages and pro-tumor (M2) macrophages. M1 macrophages are activated by GM-CSF, IFN-γ, LPS, and other cytokines. M1 macrophages, referred to as “fight” macrophages, play a significant role in producing pro-inflammatory cytokines and inducing anti-tumor immune responses (192, 193). The growth factor, GM-CSF, regulates the differentiation of DCs and macrophages (194, 195). Results from *in vivo* studies indicate that GM-CSF suppresses breast cancer growth and metastasis (196). In contrast, M2 macrophages induce tumor proliferation, therapy resistance, tumor invasion, angiogenesis, and metastasis. M2 macrophages are polarized by colony-stimulating factor 1 (CSF1), IL-13, IL-10, IL-4, TGF-β, and prostaglandin E2 (197, 198). The upregulation of CSF-1 signaling correlates with increased breast cancer progression (Figure 3C) (199). CSF1R is expressed by both M1/M2 TAMs, MDSCs, neutrophils, and DCs (200). CSF1/CSF1R signaling increases angiogenesis, cancer growth, metastases, invasion, CD8+ T cell suppression, tumor macrophage recruitment, and resistance to therapy (201, 202). CSF1 can also stimulate VEGF production (196). Blocking CSF1 in breast cancer-bearing mice reversed these effects and increased mouse survival rate (203). There are currently many therapeutic agents that target CSF1 and its receptor CSF1R, in preclinical or clinical development stages. For example, LY3022855, a CSF1R blocker used as a single agent or in combination with Durvalumab (anti-PDL1 mAb) or Tremelimumab (anti-CTLA4 mAb) for patients with a solid tumors, including breast cancers (24). Pexidartinib is another inhibitor of CSF1R that is used in combination with a microtubule inhibitor (Eribulin) for breast cancer patients (24). Anti- CSF1R (Emactuzumab) combined with Atezolizumab (anti-PDL1 mAb) are used to treat TNBC (24, 204).

## Vascular Endothelial Growth Factor A (VEGF-A)

VEGF binding to its receptors promotes the progression, proliferation, and metastasis of breast cancer (Figure 3D) (22, 205, 206). Among the five identified VEGF subfamilies (VEGF-A, VEGF-B, VEGF-C, VEGF-D, VEGF-E), VEGFA, also called VEGF, is the dominant and most researched isoform (207). VEGF isoforms bind with varying affinities to VEGFR1, VEGFR2, and VEGFR3, which mediates VEGF downstream signaling (208). VEGFA is overexpressed in several types of cancer, including breast cancer (209), and plays a vital role in angiogenesis (210). VEGF halts the differentiation and activation of DCs and promotes the exhaustion of CD8+ T cells by increasing the expression of inhibitory receptors, such as PD-1, TIM-3, LAG-3, and CTLA-4 (211). High VEGF plasma levels in breast cancer patients is associated with a significant reduction of DCs in the peripheral blood of cancer patients. The appearance of immature DCs in the blood correlates with the duration and disease stage; surgical removal of tumors showed a partial reversal of the noted effects (212). On the other hand, inhibiting VEGF increases tumor-infiltrating effector T-cells and reduces the recruitment of Tregs and MDSCs to the TME (213). Blocking VEGF stops the growth of tumor blood vessels in murine models and promotes cancer cell death and tumor-shrinkage (214). Therefore, targeting VEGF and its receptor VEGFR are key therapeutic targets for breast cancer treatment. Many angiogenesis inhibitors have been approved by the FDA, however, only a few have been tested in breast cancer patients such as bevacizumab, which binds to VEGFA and blocks its efficacy (215). Bevacizumab was the first FDA approved antiangiogenic agent (216, 217). In 2008, it was approved to be used in combination with chemotherapy to treat metastatic HER2-negative breast cancer (218). However, it showed several adverse side effects and poor overall survival, which led the FDA to revoke its approval in 2011 (219, 220). An example of a VEGFR inhibitor is DC101, a monoclonal antibody which binds to VEGFR2, and exhibits potential antiangiogenic efficacy against breast tumors in xenograft models. In another *in vivo* study, DC101 enhanced tumor-specific CD8+ T cells and accelerated tumor regression. Combining DC101 with neu-specific vaccination also suppressed tumor progression and increased the activity of CD8+ T cells (221). Ramucirumab, a VEGFR2 blocker, has shown preclinical and clinical promise in targeting breast cancer angiogenesis, growth, and metastasis (222). Axitinib is a small molecule that binds selectively to VEGFR-1,−2, and−3, and blocks their activities (223); murine studies indicate its potency in inhibiting breast cancer growth (224). However, clinical studies have only demonstrated its activity in combination with chemotherapy (paclitaxel). Sorafenib is another small molecule VEGFR blocker; reports indicate encouraging clinical trial results from the treatment of breast cancer patients. However, the utilization of sunitinib, a VEGFR inhibitor, has not shown any clinical benefit in breast cancer patients (225).

Overall, the preclinical results obtained from the use of anti-VEGF agents showed a significant decrease in tumor angiogenesis. However, the outcome of clinical trials exhibited an average response (22, 226).

## Toll-Like Receptor (TLR)

TLRs are expressed by both cancer and immune cells (227, 228). Among the thirteen TLRs (TLR1-13) that have been characterized, ten (TLR1-10) were identified in humans, six of which are expressed on the cell surface TLR (1, 2, 4, 5, 6, and 10) and four on endosomal membranes (229).

**Figure 4:**
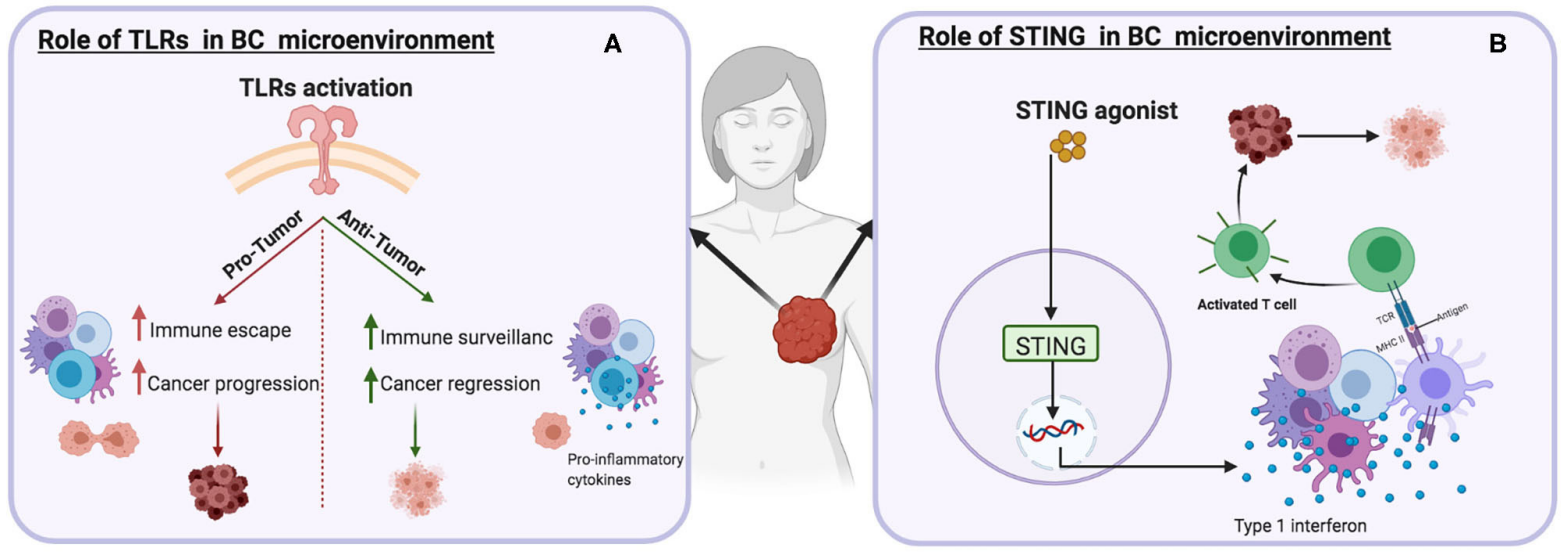
Schematic illustrations depicting the effects of different immune targets on breast cancer **(A)** Toll-like receptor(s) **(B)** Stimulator of interferon genes protein.

Several TLRs are upregulated in human breast tumors. TLR4 is the most expressed among the TLR family, on breast cancer cells (MDA-MB-231 cells). Deletion of the TLR4 gene resulted in an increase in cell death and suppression of IL-6 and IL-8 expression (230). The overexpression of TLR9 in human breast cancer enhances tumor cell invasion, which is mechanistically linked to the induction of MMP13 and COX-2 secretion (231). Various studies have reported positive correlations between TLR expression and the activation of the immune system. TLR stimulates DCs and macrophages and promotes the secretion of pro-inflammatory cytokines and the facilitation of anti-tumor immune responses (232, 233). The role of TLRs as pro-tumor agents has also been investigated (234). The function of TLRs in cancer can be described as a “double-edged sword” (Figure 4). On the one hand, agonists that bind to TLR(s) on tumor cells can promote cancer progression by promoting immune escape and cancer cell proliferation and survival. The engagement of TLR4 expressed by human breast cancer cells results in increased production immunosuppressive factors such as NO, VEGF, and MMPs, thereby promoting the tumor invasion (230, 235, 236).

On the other hand, activating TLR5 in the breast cancer mouse model resulted in anti-proliferative efficacy through the promotion of necrosis, increased neutrophil infiltration and down-regulation of cyclin B1, cyclin D1, and cyclin E2 (237). TLR3 expressed by human and mouse breast cancer cells promotes apoptosis by inducing type I IFN signaling (238). Preclinical studies have demonstrated that TLR agonists, combined with other therapeutic agents, can potentially reduce and suppress tumor progression (239, 240). The different roles of TLRs are linked to the proximal signaling pathways stimulated in cancer cells and immune cells. For example, even though TLR5 is overexpressed in both gastric and breast cancers, it has opposite effects as it suppresses the proliferation of breast cancer and induces the growth of gastric cancer cells (237, 241). Many TLR agonists have been investigated for clinical use. The TLR5 agonist, flagellin, suppresses breast cancer by induction of caspase-1 activation-dependent pyroptosis. It also enhances the expression of granzyme B, TNF-α, and IFN-γ in CD8+ T cells (242, 243). The TLR3 ligand, poly-AU, increases the survival rate in patients with TLR3-positive breast cancer (244). Imiquimod is a well-tolerated TLR7 agonist that can promote the rejection of immune-mediated skin metastasis in breast cancer patients (245) 852A is another TLR7 agonist used for the treatment of metastatic breast cancer patients (240).

## Stimulator of Interferon Genes Protein (STING)

Various studies have suggested that STING (stimulator of interferon (IFN) genes) expression is not only confined to innate and adaptive immune cells (246–248), but is also expressed in various tumors, including breast cancer (249). STING stimulators have shown great potential for activating immune cells, enhancing anti-tumor immunity by inducing a variety of pro-inflammatory cytokines and chemokines (246, 247, 250), priming and activation of T cells (251), enhancement of antigen presentation, promotion of cancer cell death, inducing the recognition and apoptosis of cancer cells by T cells (249, 252, 253). A previous study has revealed the role of STING in promoting death in 4T1 breast cancer cells by increasing the caspase-3 pathway cascade (249). Similarly, the overexpression of STING in two breast cancer cell lines, T47D or MCF-7 has been shown to increase caspase 3 and/or 7 activity (252). The deletion of STING expressed by melanoma cell lines results in the suppression of cytokines (IFN-γ) and chemokines (CCL5 and CXCL10) production (254). Furthermore, STING knockout mice exhibit reduced NK cell responses by mediating the downregulation of perforin, granzyme B, and IFN-γ (253, 254). Numerous STING stimulators are now under clinical investigation for the treatment of various types of cancers. The utilization of ADU-S100 (MIW815), a STING agonist, is currently being tested in combination with anti-PD-1 (spartalizumab) for the treatment of patients with solid tumors, including PD-1-naïve TNBC (255).

## Conclusions

Following several years of preclinical and clinical research, our understanding of how the immune system responds to cancer has increased. The limited success of immune checkpoints, like CTLA-4 or PD-1, in clinical trials for breast cancer patients, has prompted research to find alternative targets. Many new emerging data reported novel pathways that stimulate immune responses against breast tumors. These newly discovered pathways are likely to be the future targets of breast cancer immunotherapy.

## Author Contributions

YT and IO drafted the manuscript. KB, AS, and SE edited and modified the manuscript. All authors contributed to the article and approved the submitted version.

## Conflict of Interest

The authors declare that the research was conducted in the absence of any commercial or financial relationships that could be construed as a potential conflict of interest.
